# Epilepsia partialis continua as the presenting manifestation of Creutzfeldt–Jakob disease: A video‐polygraphic clinical vignette

**DOI:** 10.1002/epd2.70238

**Published:** 2026-04-03

**Authors:** Roberta Cutellè, Angelo Pascarella, Ada Santoro, Vittoria Cianci, Anna Mammì, Giovanbattista Gaspare Tripodi, Caterina Prestandrea, Sara Gasparini, Edoardo Ferlazzo

**Affiliations:** ^1^ Department of Medical and Surgical Sciences Magna Græcia University Catanzaro Italy; ^2^ Regional Epilepsy Centre, Great Metropolitan “Bianchi‐Melacrino‐Morelli” Hospital Reggio Calabria Italy; ^3^ Neurology Unit Great Metropolitan “Bianchi‐Melacrino‐Morelli” Hospital Reggio Calabria Italy

**Keywords:** focal motor status epilepticus, lateralized periodic discharges, myoclonus, polygraphy, prion disease, Syndrome: Epilepsia partialis continua, Etiology: Degenerative disease, Phenomenology: Myoclonic seizure, status epilepticus (non‐convulsive)

Epilepsia partialis continua (EPC) is an exceptionally rare presenting feature of Creutzfeldt–Jakob disease (CJD).^1^ We describe a 48‐year‐old man with familial CJD (fCJD) who presented with EPC as the initial manifestation. Video‐polygraphic recordings demonstrated a temporal correlation between lateralized periodic discharges (LPDs) and the patient's left upper limb jerks, confirming their cortical origin. To our knowledge, this represents the first video‐polygraphically documented case of EPC in CJD, highlighting the diagnostic value of detailed neurophysiological assessment. Relevant literature is reviewed in Table 1.

**TABLE 1:** Main clinical, neurophysiological, and neuroradiological findings of reported cases of patients with EPC at the onset of CJD.

References	Age at onset, gender	Clinical manifestations at disease onset	EPC semiology	EEG	MRI	EPC treatment and outcome	Prognosis	Diagnosis
Lee et al., 2000^5^	42, M	Forgetfulness, unsteady gait, involuntary movement of the right arm	Semirhytmic clonic movement of the right arm and hand	Left hemispheric LPDs	FLAIR hyperintensity in the occipital cortex	Improvement of involuntary movement (treatment not reported)	Not reported	Definite sCJD
Parry et al., 2001^6^	67, F	Involuntary movement of the left upper limb	Intermittent contractions of hand and forearm flexor muscles	Intermittent low amplitude slow waves over the right parietal region	Not performed	(1) PHT, CZP with improvement for 2 weeks; (2) VPA e LTG, (PHT withdrawal) with EPC worsening	Death after 6 weeks from onset	Definite sCJD
Donmez et al., 2005^7^	47, F	Anxiety, unsteadiness, concentration difficulty, generalized chorea, focal dystonia, myoclonic jerks on the right hand with progression to EPC within 5 days	Myoclonic jerks on the right hand	Diffuse slowness of basal rhythm marked on left side and biphasic sharp waves correlating with myoclonic jerks.	Basal ganglia DWI and FLAIR hyperintensity	VPA with no response	Death after 24 weeks	Probable sCJD
Lowden et al., 2008^1^	49, F	Left upper extremity weakness, left upper extremity jerks	Continuous, rhythmical left upper limb jerks	LPDs on right centroparietal region	Bilateral caudates, putamen and thalami pulvinars T2, DWI and FLAIR hyperintensity	PB, LEV, LTG, ZNS, PHT (not reported improvement)	Death after 11 weeks	fCJD
Taskiran et al., 2010^8^	70, M	Speech difficulty, inability to walk, numbness and jerks in the right hand	Rhythmical right hand jerks, subsequent extending proximally and in the right lower limb	LPDs on the fronto‐centro‐temporal region	Bilateral caudate nuclei (left >right), left frontal temporal and parietal cortex DWI hyperintensity	LEV with some improvement	Death after 8 weeks	Probable sCJD
Yang et al., 2018^9^	62, M	Numbness and rhythmic jerking‐tremors in the left hand subsequently extending to left arm or leg	Rhythmic jerking‐tremors in the left hand extending occasionally to the left arm and leg	Independent epileptic foci at right central, frontal and occipital areas	Bilateral frontoparietal, right insular and occipital cortex, bilateral caudate nuclei and putamen DWI hyperintensity	Various ASMs: partial response with VPA and TPM	Death after 20 weeks	Probable sCJD
Cunha et al., 2021^10^	70, M	Left‐sided involuntary movements	Left body clonic movement	GPDs	Basal ganglia, cortex and cerebellum DWI, T2 and FLAIR hyperintensity	LEV, VPA, LCM, PB, CZP without improvement	Death after 13 weeks	Definite sCJD

*Note*: References are ordered by publication date.

Abbreviations: ASM, antiseizure medications; CJD, Creutzfeldt–Jacob disease; CZP, clonazepam; EPC, Epilepsia Partialis Continua; fCJD, familial CJD; LCM, lacosamide; LEV, levetiracetam; LTG, lamotrigine; PHT, phenytoin; sCJD, sporadic CJD; PB, phenobarbital; TPM, topiramate; VPA, valproic acid; ZNS, zonisamide.

CJD is a fatal neurodegenerative disorder caused by accumulation of misfolded prion proteins in the central nervous system.^2^ It primarily affects individuals aged 55–75 years, with an annual incidence of 1–2 cases per million worldwide. CJD occurs in sporadic, genetic (familial) or acquired forms; fCJD accounts for ≈10%–15% and is mostly associated with the E200K PRNP mutation.^2^ Clinically, CJD is characterized by rapidly progressive dementia, myoclonus, cerebellar ataxia, visual disturbances and pyramidal or extrapyramidal signs. Epileptic seizures are uncommon and rarely represent the initial manifestation.^3^ EPC is a rare subtype of focal motor status epilepticus, characterized by repetitive, arrhythmic, stereotyped muscle jerks persisting over long periods.^4^ Its occurrence as the initial manifestation of CJD is exceedingly rare, with only seven cases reported.^1^, ^5^, ^6^, ^7^, ^8^, ^9^, ^10^

Our previously healthy patient presented with involuntary movements of the left upper limb and progressive gait unsteadiness over 2 weeks. His father had died from E200K‐related fCJD. Neurological examination showed mild gait ataxia and shock‐like involuntary movements of the left upper limb. Neuropsychological evaluation disclosed preserved cognitive functions (MMSE: 26). Blood tests and cerebrospinal fluid analysis were unremarkable. Brain MRI revealed restricted diffusion with corresponding T2/FLAIR hyperintensity involving the right caudate nucleus, anterior lentiform nucleus, cingulate gyrus, insula, and less prominently the right frontal, parietal, and temporal cortices (Figure S1). Video‐polygraphy (EEG, EKG and electromyography of left upper limb and facial muscles) revealed LPDs over the right fronto‐central‐temporal regions. Each spike was time‐locked with short‐lasting (<50 msec) myoclonic jerks of the left upper limb (Video 1), consistent with EPC. Treatment with multiple antiseizure medications (clonazepam, levetiracetam, brivaracetam) was ineffective. The patient's condition rapidly deteriorated over few weeks, with cognitive decline, severe ataxia, generalized tonic–clonic seizures, akinetic mutism. At this time, EEG showed generalized periodic discharges (GPDs) occurring at 1–2 s intervals. The patient died 45 days after admission. Genetic testing confirmed the E200K mutation; PRNP codon 129 polymorphism was not assessed.

**VIDEO 1:** Video‐polygraphic recording. The video shows continuous, arrhythmic (1–2 Hz), myoclonic jerks of the patient's distal left upper limb. Polygraphic recording (EMG: deltoid and wrist flexors/extensors muscles) revealed lateralized periodic discharges over the right fronto‐central‐temporal regions (red box) time‐locked with brief (<50 ms) EMG burst in the left wrist flexor and extensor muscles (blue arrow), in keeping with EPC. EEG montage: bipolar longitudinal; settings: high‐frequency filter at 30 Hz, low‐frequency filter at 1.6 Hz, sensitivity at 100 μV/mm, time base at 15 s (seconds 0 to 25) and 10 s (seconds 26 to 41). d, dexter (right); DEL, deltoid muscle; ECG, electrocardiogram; EST, extensor muscles; FLX, flexor muscles; MKR, marker (each complete oscillation cycle corresponds to 1 s); s, sinister (left). Video content can be viewed at https://onlinelibrary.wiley.com/doi/10.1002/epd2.70238.

Epileptic seizures occur in ≈15% of CJD patients during the disease course, being the presenting symptoms in only 3% of sCJD.^2^, ^3^ EPC is even rarer, with only seven reported patients^1^, ^5^, ^6^, ^7^, ^8^, ^9^, ^10^ and only one genetically confirmed fCJD.^10^ EPC in a previously healthy subject usually prompts investigation for alternative diagnosis, including stroke, Rasmussen encephalitis, tumor.^5^ In our patient, the characteristic DWI/FLAIR cortical and basal ganglia involvement, together with the positive family history, strongly supported the fCJD suspicion, subsequently confirmed genetically. No prior study has provided video‐polygraphic documentation of EPC in CJD. In our patient, polygraphic recordings showed distal left upper limb EMG discharges lasting <100 ms time‐locked to right fronto‐central‐temporal LPDs, providing clear electrophysiological evidence of EPC.^4^ LPDs are not specific to CJD, with GPDs representing the most characteristic finding reported in intermediate disease stages in sCJD.^3^, ^11^ Nevertheless, LPDs have been documented in early CJD^2^ and in 3/7 of reported patients with EPC, including one with fCJD.^1^, ^5^, ^8^ LPD lateralization typically mirrors cortical MRI abnormalities, correlates with contralateral focal motor signs and frequently evolves into GPDs with disease progression, as prion aggregates spread from restricted cortical areas to diffuse bilateral cortical regions.^3^ Patients with EPC associated with CJD appear to have a worse prognosis than those with typical presentations.^8^ Review of reported cases shows survival after EPC onset ranging from a few weeks to a few months, with a median of approximately 2–3 months,^1^, ^6^, ^7^, ^8^, ^9^, ^10^ considerably shorter than the usual 6–12 months in sporadic CJD and 5–9 months in E200K‐related fCJD. Our patient's rapid clinical course is fully consistent with these observations, supporting evidence that EPC at disease onset may represent a marker of an especially aggressive phenotype, likely reflecting early cortical involvement. Nevertheless, the more aggressive course may be a consequence of epilepsy itself, including potential adverse effects of anti‐seizure medications and an increased risk of sudden unexpected death in epilepsy (SUDEP).^8^ Additionally, the possible influence of PRNP codon 129 polymorphism on the clinical phenotype should be taken into account.^12^

In conclusion, EPC may represent an early manifestation of CJD. A high index of suspicion is warranted when EPC occurs in the context of rapidly progressive neurological symptoms.

## FUNDING INFORMATION

Work supported by #NEXTGENERATIONEU (NGEU) and funded by the Ministry of University and Research (MUR), National Recovery and Resilience Plan (NRRP), project MNESYS (PE0000006) – A Multiscale integrated approach to the study of the nervous system in health and disease (DN. 1553 11.10.2022).

## CONFLICT OF INTEREST STATEMENT

The authors have no competing interests to declare that are relevant to the content of this article.

**Test yourself**

1. Which percentage of patients with sporadic CJD present with epileptic seizures as an early manifestation?
  - 1%
  - 3%
  - 10%
  - 15%
  - 25%
2. In CJD, lateralized periodic discharges are most frequently observed:
  - During late stages, after akinetic mutism develops
  - Only in familial forms linked to E200K mutation
  - Mainly in the early phases of the disease
  - Exclusively in the occipital regions
  - Exclusively in the parietal regions
3. Which is the prognosis of patients with CJD presenting with Epilepsia Partialis Continua (EPC) at disease onset?
  - Very mild course with death occurring after 5 years from EPC onset
  - Very mild course with death occurring after 2 years from EPC onset
  - Aggressive disease course
  - Death within 2 days from EPC onset
  - Death within 1 day from EPC onset
*Answers may be found in the* *supporting information*.

## Supporting information


Figure S1

Data S1

Data S2

## Data Availability

The data that support the findings of this study are available from the corresponding author upon reasonable request.
