# Supplementary figures and images for: Epilepsia partialis continua as the presenting manifestation of Creutzfeldt–Jakob disease: A video‐polygraphic clinical vignette
Source: Epileptic Disord. 2026 Apr 3;28(3):920–4. doi: 10.1002/epd2.70238 (PMC13276695; doi:10.1002/epd2.70238)

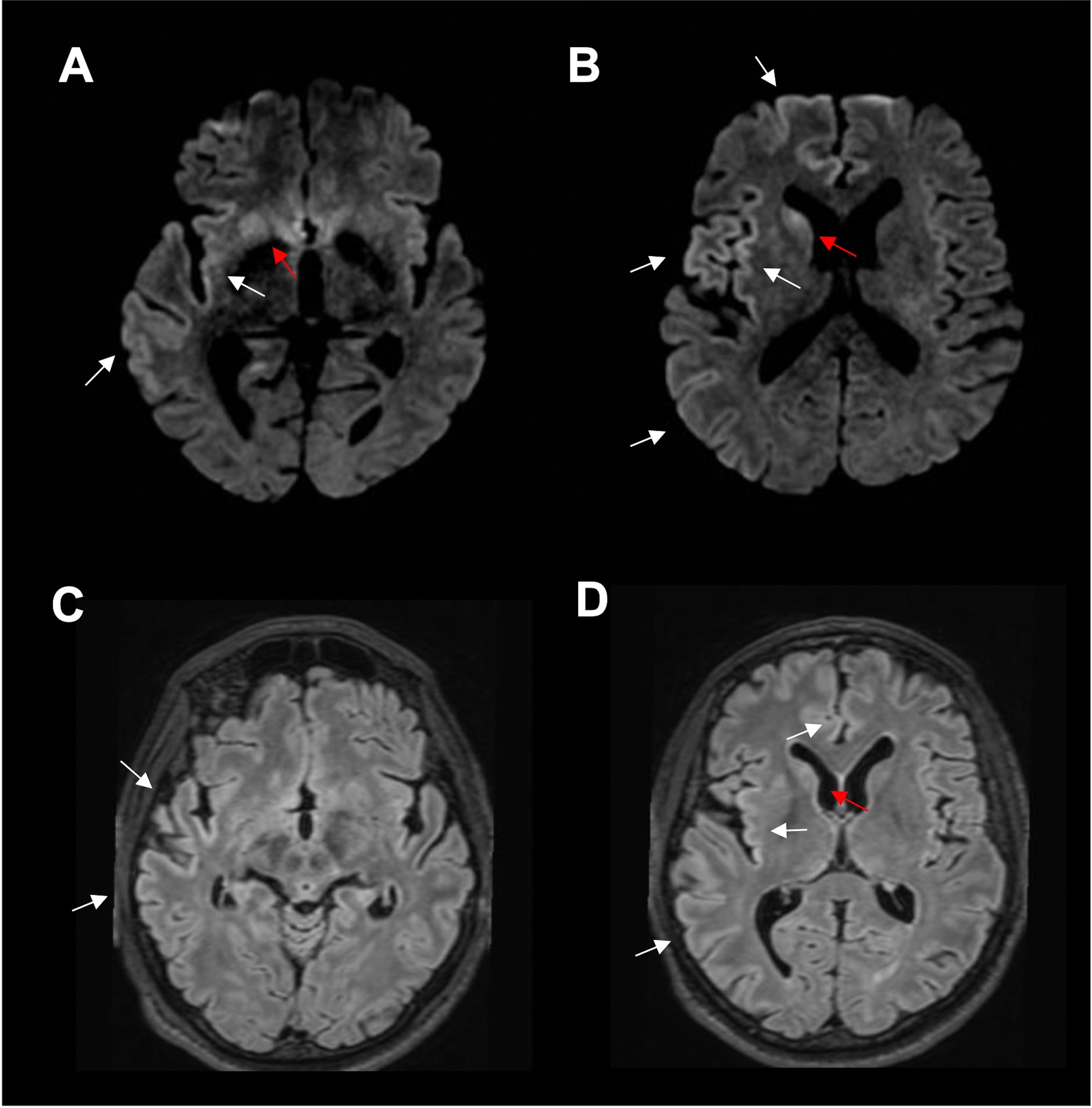

Supplement: Supplementary file 2 — Figure S1 [file EPD2-28-920-s001.jpg]
