# Supplementary material for: Epilepsia partialis continua as the presenting manifestation of Creutzfeldt–Jakob disease: A video‐polygraphic clinical vignette
Source: Epileptic Disord. 2026 Apr 3;28(3):920–4. doi: 10.1002/epd2.70238 (PMC13276695; doi:10.1002/epd2.70238)
Supplement: Supplementary file 3 — Data S1 [file EPD2-28-920-s002.pptx]

## Slide 1
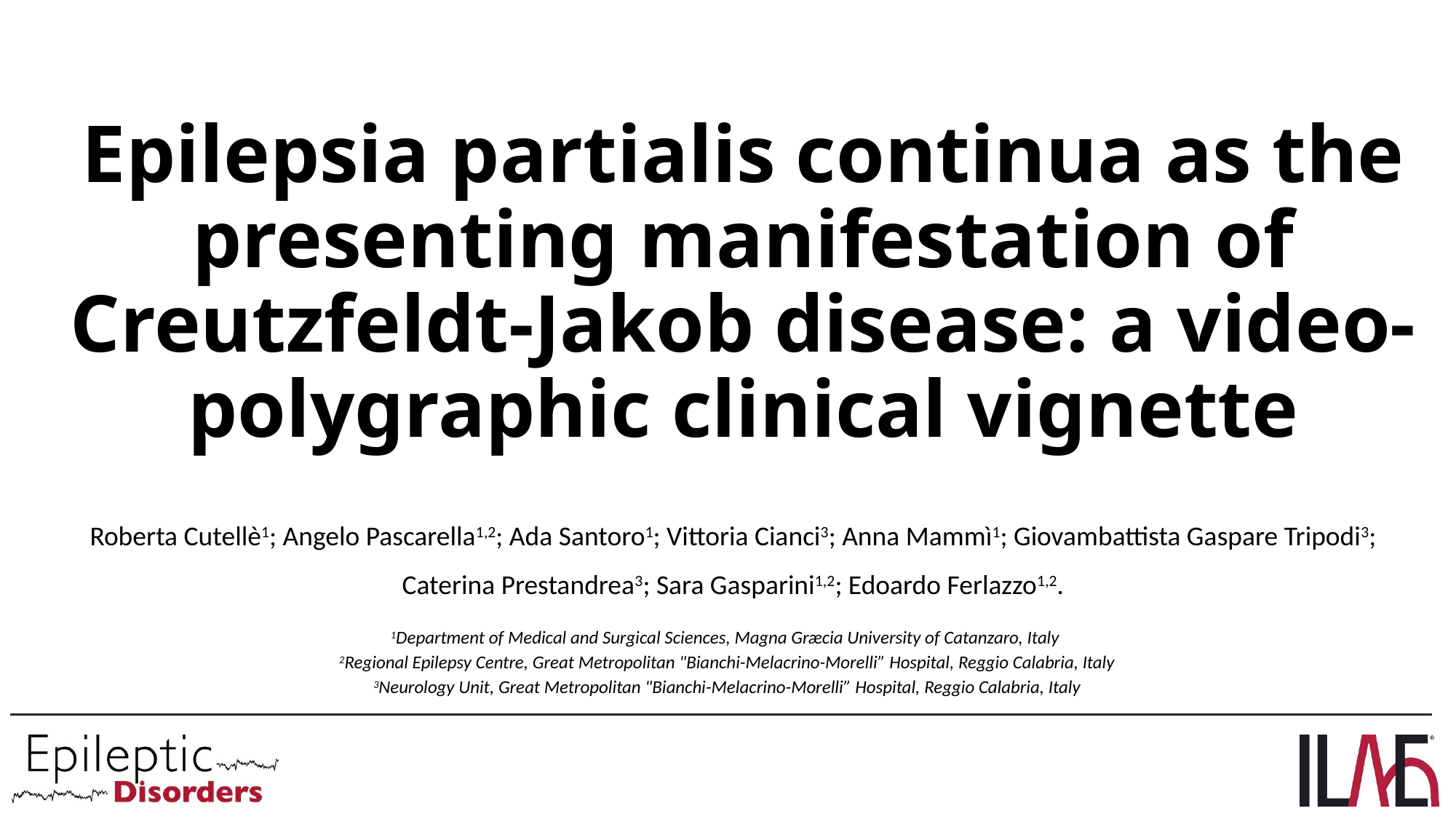

# Epilepsia partialis continua as the presenting manifestation of Creutzfeldt-Jakob disease: a video-polygraphic clinical vignette
Roberta Cutellè1; Angelo Pascarella1,2; Ada Santoro1; Vittoria Cianci3; Anna Mammì1; Giovambattista Gaspare Tripodi3; Caterina Prestandrea3; Sara Gasparini1,2; Edoardo Ferlazzo1,2.
1Department of Medical and Surgical Sciences, Magna Græcia University of Catanzaro, Italy
2Regional Epilepsy Centre, Great Metropolitan "Bianchi-Melacrino-Morelli” Hospital, Reggio Calabria, Italy
3Neurology Unit, Great Metropolitan "Bianchi-Melacrino-Morelli” Hospital, Reggio Calabria, Italy

## Slide 2
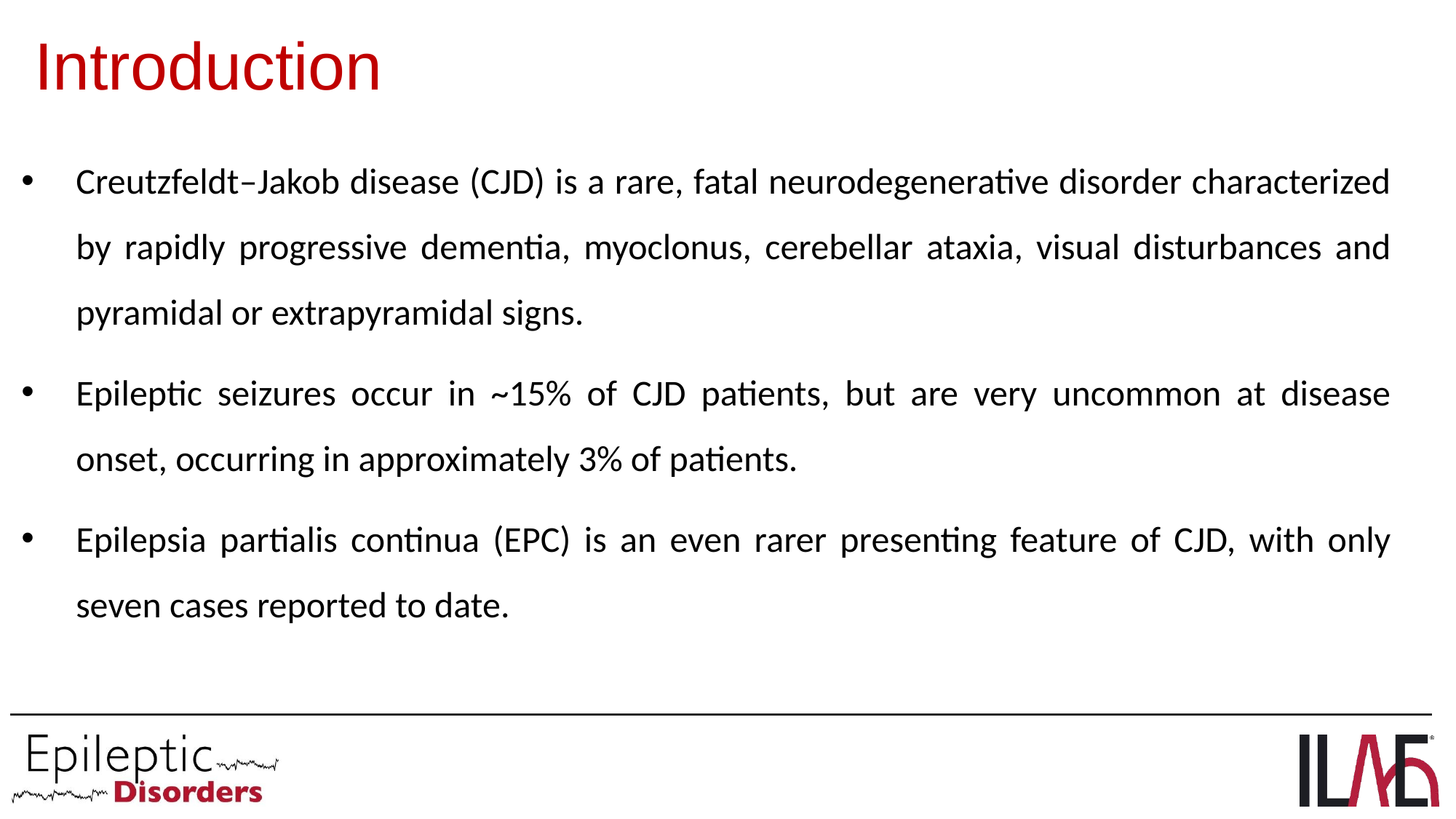

Introduction
Creutzfeldt–Jakob disease (CJD) is a rare, fatal neurodegenerative disorder characterized by rapidly progressive dementia, myoclonus, cerebellar ataxia, visual disturbances and pyramidal or extrapyramidal signs.
Epileptic seizures occur in ~15% of CJD patients, but are very uncommon at disease onset, occurring in approximately 3% of patients.
Epilepsia partialis continua (EPC) is an even rarer presenting feature of CJD, with only seven cases reported to date.

## Slide 3
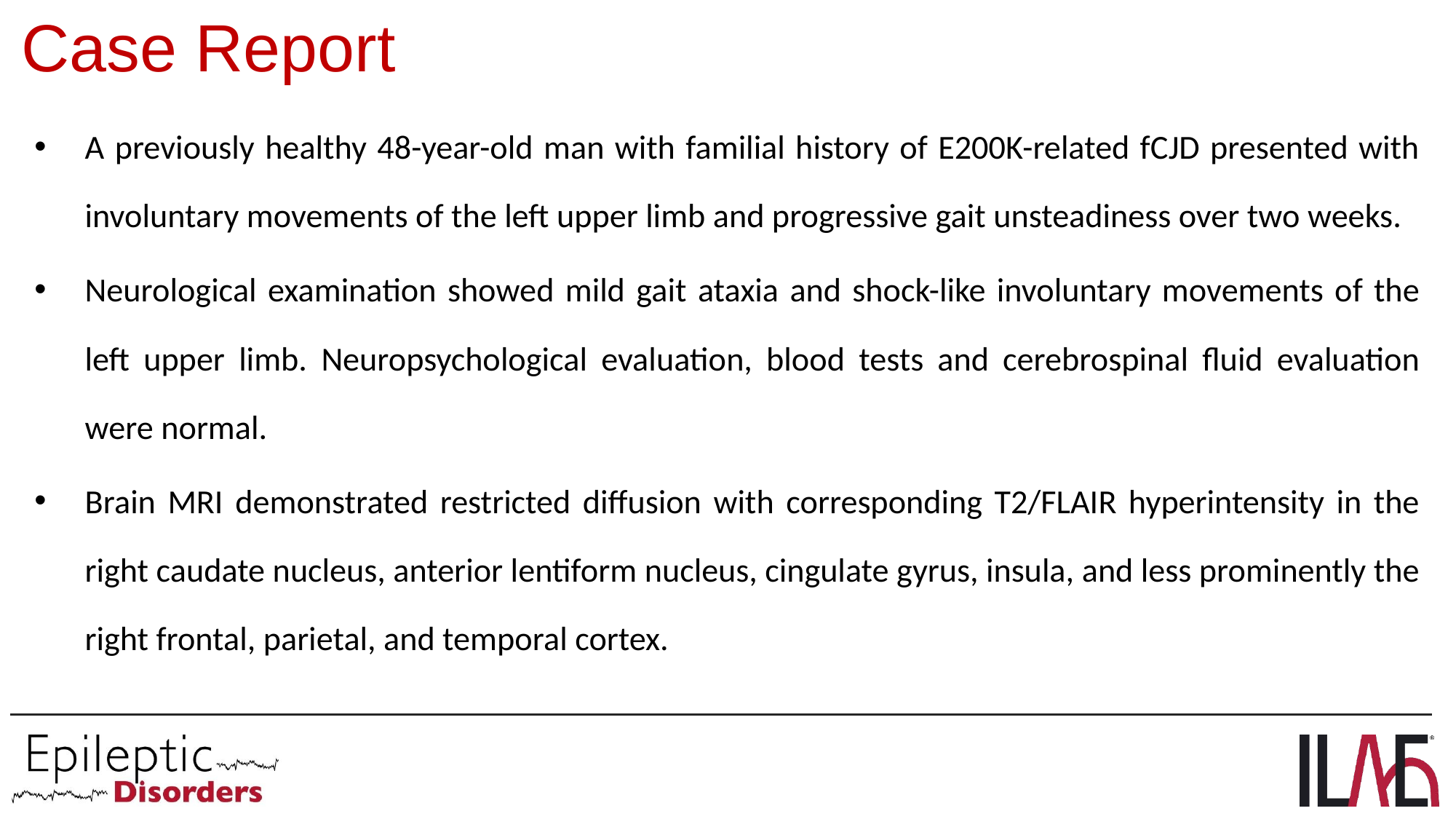

Case Report
A previously healthy 48-year-old man with familial history of E200K-related fCJD presented with involuntary movements of the left upper limb and progressive gait unsteadiness over two weeks.
Neurological examination showed mild gait ataxia and shock-like involuntary movements of the left upper limb. Neuropsychological evaluation, blood tests and cerebrospinal fluid evaluation were normal.
Brain MRI demonstrated restricted diffusion with corresponding T2/FLAIR hyperintensity in the right caudate nucleus, anterior lentiform nucleus, cingulate gyrus, insula, and less prominently the right frontal, parietal, and temporal cortex.

## Slide 4
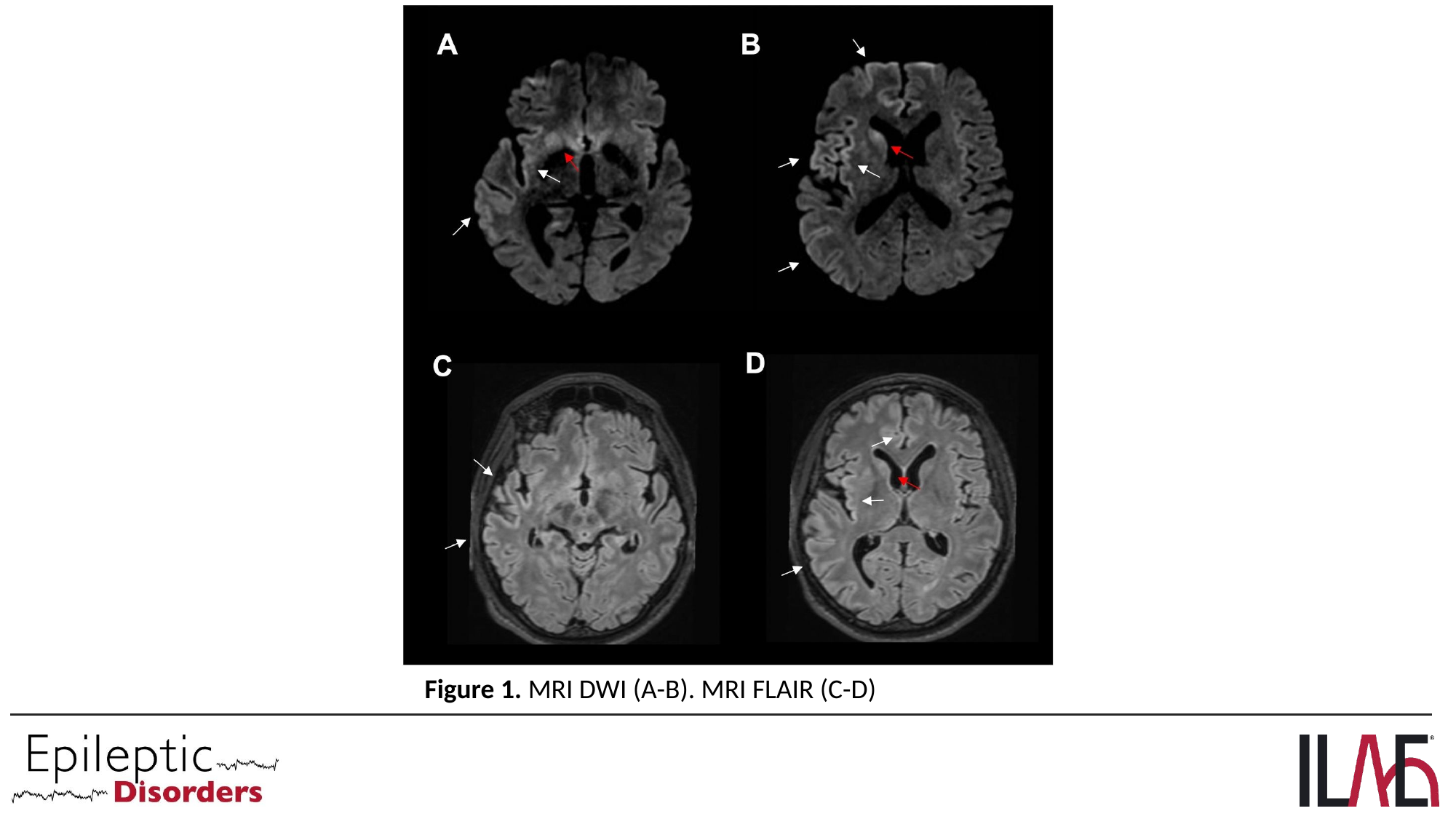

Figure 1. MRI DWI (A-B). MRI FLAIR (C-D)

## Slide 5
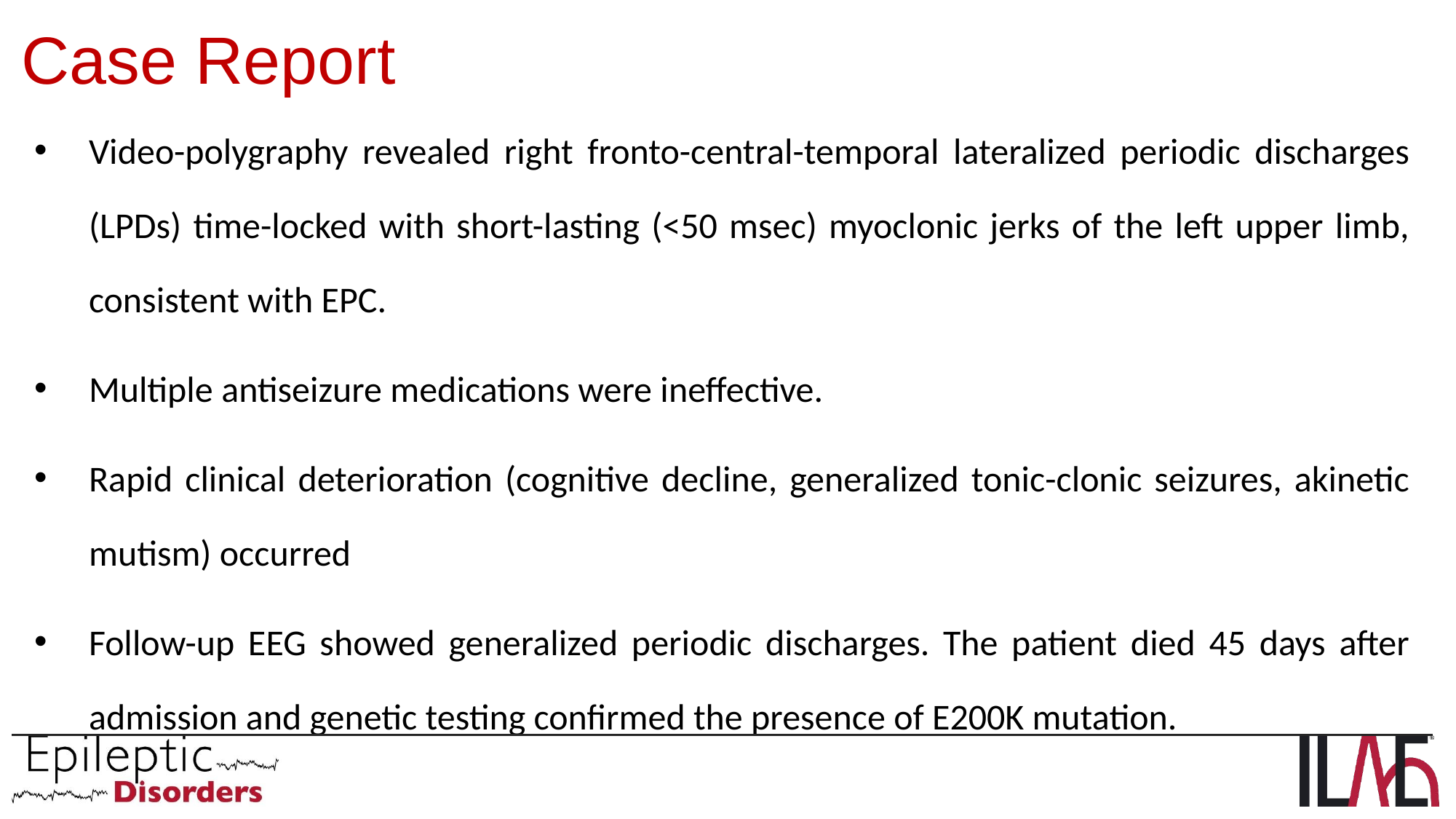

Case Report
Video-polygraphy revealed right fronto-central-temporal lateralized periodic discharges (LPDs) time-locked with short-lasting (<50 msec) myoclonic jerks of the left upper limb, consistent with EPC.
Multiple antiseizure medications were ineffective.
Rapid clinical deterioration (cognitive decline, generalized tonic-clonic seizures, akinetic mutism) occurred
Follow-up EEG showed generalized periodic discharges. The patient died 45 days after admission and genetic testing confirmed the presence of E200K mutation.

## Slide 6
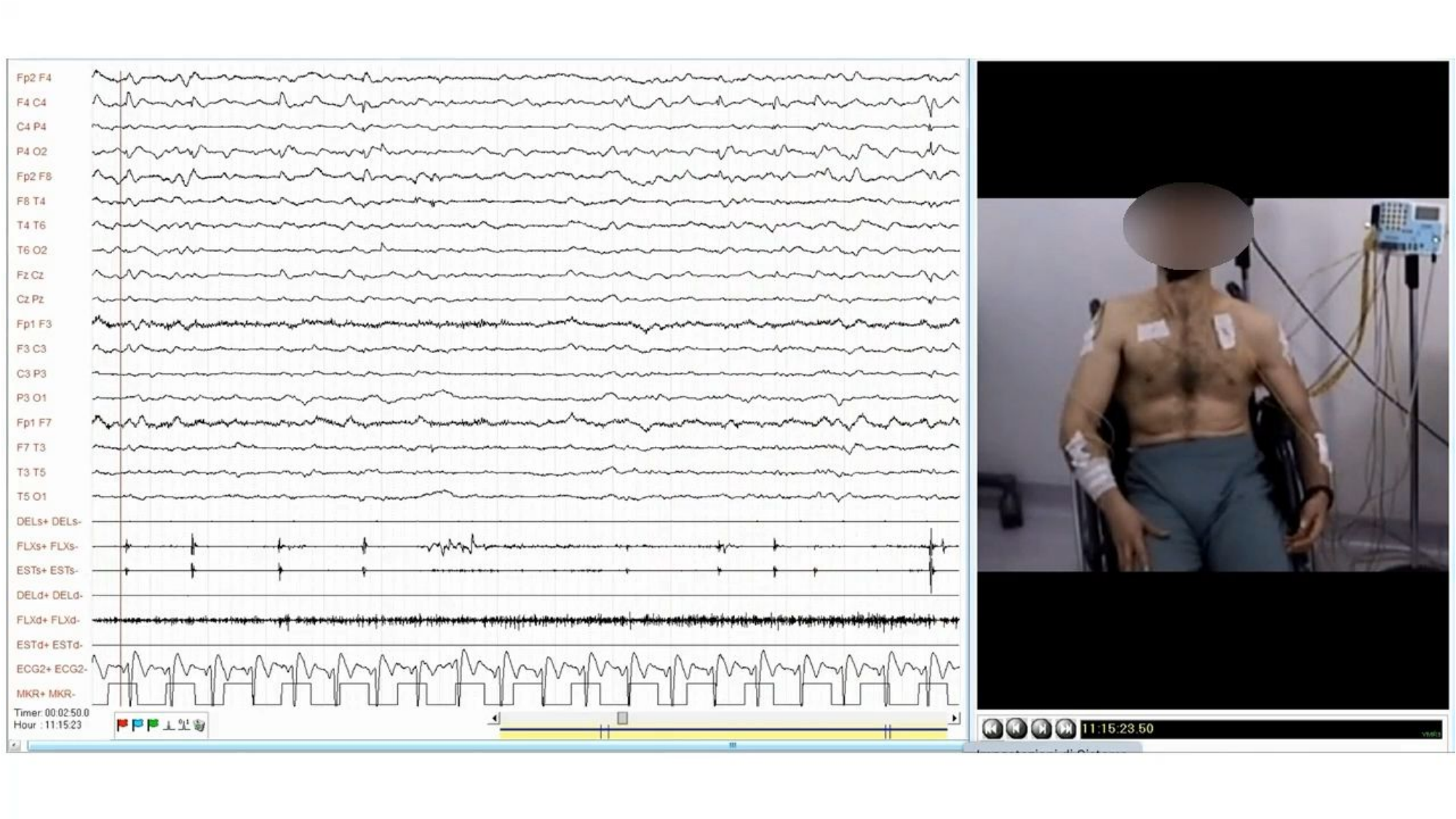

## Slide 7
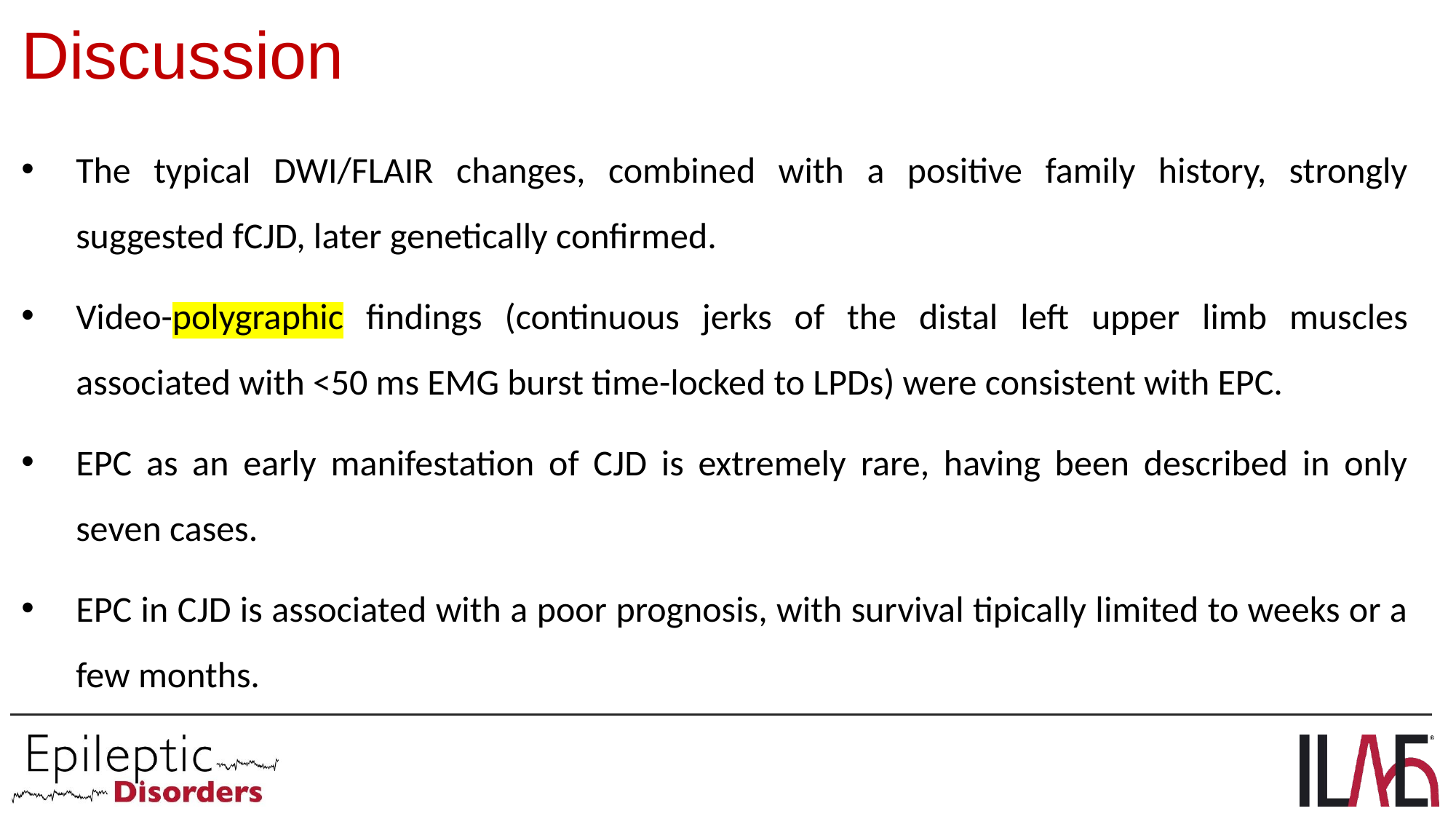

Discussion
The typical DWI/FLAIR changes, combined with a positive family history, strongly suggested fCJD, later genetically confirmed.
Video-polygraphic findings (continuous jerks of the distal left upper limb muscles associated with <50 ms EMG burst time-locked to LPDs) were consistent with EPC.
EPC as an early manifestation of CJD is extremely rare, having been described in only seven cases.
EPC in CJD is associated with a poor prognosis, with survival tipically limited to weeks or a few months.

## Slide 8
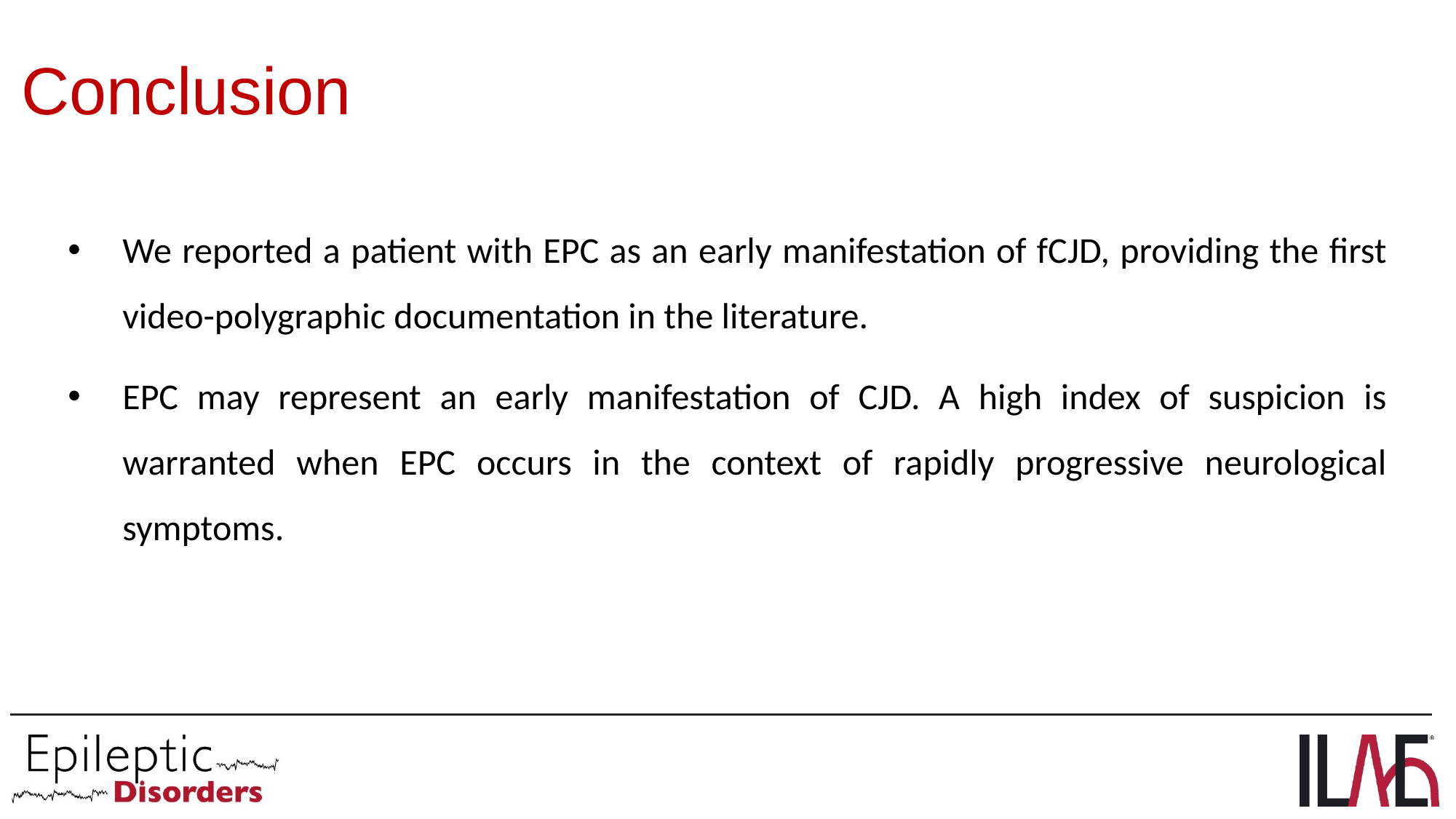

Conclusion
We reported a patient with EPC as an early manifestation of fCJD, providing the first video-polygraphic documentation in the literature.
EPC may represent an early manifestation of CJD. A high index of suspicion is warranted when EPC occurs in the context of rapidly progressive neurological symptoms.
