# Supplementary material for: Epilepsia partialis continua as the presenting manifestation of Creutzfeldt–Jakob disease: A video‐polygraphic clinical vignette
Source: Epileptic Disord. 2026 Apr 3;28(3):920–4. doi: 10.1002/epd2.70238 (PMC13276695; doi:10.1002/epd2.70238)
Supplement: Supplementary file 4 — Data S2 [file EPD2-28-920-s003.docx]

Answers

(1) Correct answer: (b) 3%

(2) Correct answer: (c) Mainly in the early phases of the disease

(3) Correct answer: (c) Aggressive disease course
